# *APOE* ε4 allele accelerates age-related multi-cognitive decline and white matter damage in non-demented elderly

**DOI:** 10.18632/aging.103367

**Published:** 2020-06-22

**Authors:** Jinping Sun, Zhibao Zhu, Kewei Chen, Dongfeng Wei, Xin Li, He Li, Junying Zhang, Xiaochun Chen, Yaojing Chen, Zhanjun Zhang

**Affiliations:** 1 Department of Neurology, The Affiliated Hospital of Qingdao University, Qingdao, China; 2 State Key Laboratory of Cognitive Neuroscience and Learning, Beijing Normal University, Beijing, China; 3 BABRI Centre, Beijing Normal University, Beijing, China; 4 Department of Neurology, and Fujian Key Laboratory of Molecular Neurology, Fujian Medical University Union Hospital, Fuzhou, China; 5 Banner Alzheimer's Institute, Phoenix, AZ 85006, USA; 6 Institute of Basic Research in Clinical Medicine, China Academy of Chinese Medical Sciences, Beijing, China

**Keywords:** Alzheimer’s disease, apolipoprotein E, aging, cognition, white matter

## Abstract

Advanced age and apolipoprotein E (*APOE*) ε4 allele are both associated with increased risk of the Alzheimer’s disease (AD). However, the extent of the joint contribution of *APOE* ε4 allele and age on the brain white matter integrity, cognition and their relationship are unclear. We assessed the age-related variation differences of major cognitions in 846 non-demented elderly, and brain major white matter tracts in an MRI sub-cohort of 111 individuals between ε4 carriers and noncarriers. We found that: (i) carriers showed a steeper age-related decline after age 50 in general mental status, attention, language, and executive function and performed worse than noncarriers at almost all ages; (ii) main effect of age on anterior fibers, but main effect of *APOE ε*4 on posterior fibers, and the interactive effect of them existed on anterior and posterior fibers; (iii) carriers showed an accelerated age-related integrity reduction of these fibers compared to noncarriers who had a slight decrease but not significant; and (iv) significant associations of the higher white matter integrity with better multi-cognitive performance in old ε4 carriers. Overall, combining *APOE* status with age may be useful in assessing possible mechanisms of disease development in AD.

## INTRODUCTION

Alzheimer’s disease (AD) is characterized by complicated pathologic process and lengthy preclinical and clinical courses. Abundant evidence supports that the intervention in advanced, later stage of AD is ineffective. Consequently, investigations of preclinical AD phase have attracted more attention. Viewed by many, AD is a severe neurodegenerative disease caused by or related to multiple genetic and environmental factors [1]. Among these risk factors, the apolipoprotein E (*APOE*) ε4 allele is a well-established one. Comparing with ε4 allele noncarriers, individuals carrying one copy of ε4 allele had 2- to 3-fold, individuals carrying two copies of ε4 allele had about 12-fold AD risk [2]. A previous longitudinal study has demonstrated that cognitive normal ε4 carriers started age-related memory decline before 60 years old [3]. On the other hand, increasing age has always been the greatest risk factor of AD [4] regardless of genetic makeup though influence of which on aging has been an interesting research topic for many. Therefore, the illustration of the joint impact of age and *APOE* ε4 allele on different domains of advanced cognitive function and its potential neuromechanism have become the focus of many researchers.

As the most important apolipoprotein in the brain, the ApoE protein is the crucial carrier of cholesterol, which played an important role in the formation, development and repair of myelin, neuron membrane and axon. The microstructural damage of white matter is a significant independent factor of AD [5–7]. Mounting evidence showed that the ApoE protein is related with axonal degeneration and structural damage in microtubules [8, 9], therefore the *APOE* polymorphism may be participated in white matter integrity. The older ages have increased level of lipid peroxidation and myelin breakdown [10] compare to the younger ones in healthy individuals, while AD patients have more myelin breakdown than normal control [11]. The association between *APOE* status and the severity and rate of myelin breakdown may suggest that *APOE* genotype contributes to the onset of AD [12]. Bartzokis et al.’s later study proved that *APOE* genotype caused age-related slowing in cognitive processing speed by impacting myelin breakdown, and eventually shifted the age at onset of cognitive decline [13]. One recent study showed that relative to ε3 homozygotes, ε4 carriers showed faster executive function decline with age, even after exclusion of individuals with evidence of cognitive impairment [14].

Previous studies have suggested that cognitive measures and brain indices are strongly influenced by genetic factors [15], and such impact continuously was enhanced with aging [16, 17]. A growing body of evidence has also been supporting that *APOE* ε4 impairs the integrity of white matter (WM) fibers [18]. Advanced age is an unavoidable factor in AD. Therefore, identifying joint contribution of *APOE* ε4 allele and age on the brain and cognition could have a profound impact on our understanding of the underlying process that influences brain function in disease. Operto et al. found a significant negative interaction between the number of APOE ε4 alelles and age in extensive bilateral regions of the WM in both the recessive and the additive contrast [19]. However, does the APOE ε4 allele moderate age-related changes in WM fibers integrity? Furthermore, what is the effect of APOE ε4 - age interactions on the white matter - cognitive relationship? In this study, we examined the effects of advancing age on multi-cognition domains and white matter integrity in non-demented *APOE* ε4 carriers and noncarriers. Our goals were to (i) document age-related changes in different cognitive domains and white matter integrity in ε4 carriers and noncarriers respectively, (ii) for each cognitive function, further estimate the age trajectories by using best-fitting regression models for carriers and noncarriers respectively, (iii) determine if gene-by-aging interactions influence the relationship between cognition and white matter integrity.

## RESULTS

### Cognitive performance change with age

Characteristics and neuropsychological test scores of two groups are shown in Table 1. Age ranged from 48 to 87 years, with a mean of 64.90±7.39 for carriers and 65.18±7.34 for noncarriers. No differences in gender, education or mild cognitive impairment (MCI) proportion were found between carriers and noncarriers. There also no differences in hypertension, hyperlipidemia, diabetes, coronary heart disease, cerebrovascular disease, and family history of dementia proportion between APOE the performance of ε4 carriers in the Mini-Mental State Examination (MMSE) was significantly worse than that of non-carriers (p=0.001). Among the cognitive scores, all showed a significant age effect (p<0.05). Moreover, the *APOE* ε4 allele had an interactive effect with age significantly on MMSE (p=0.011) and visuo-spatial ability (p=0.006), and language (p=0.028).

**Table 1:** Demographic characteristics and neuropsychological test of all sample participants.

	***APOEε*4 carriers (n=129)**	***APOEε*4 noncarriers (n=717)**	**Main *APOEε*4 effect**	**Main age effect**	**Age×*APOE* ε4 interaction**
Male/Female	47/82	264/453	1^a^		
Age (years)	64.90±7.39	65.18±7.34	0.69		
Education (years)	11.08±3.61	10.82±3.67	0.47		
MCI (Y/N)	23/106	97/620	0.22 ^a^		
Hypertension (%)	51 (39.5%)	296 (41%)	0.83 ^a^		
Hyperlipidemia (%)	69(53.4%)	327 (45.6%)	0.205 ^a^		
Diabetes (%)	12 (9.3%)	116 (16.2%)	0.107 ^a^		
Coronary heart disease (%)	7 (5.4%)	82 (11.4%)	0.112 ^a^		
Cerebrovascular disease (%)	15 (11.6%)	86 (12%)	1 ^a^		
Family history of dementia (%)	3 (2.3%)	10 (13.9%)	0.332 ^a^		
MMSE	27.29±2.25	27.66±1.96	0.001	<0.0001	0.011
Memory	-0.069±0.75	0.012±0.74	0.063	<0.0001	0.144
Visuo-spatial ability	-0.053±0.91	0.003±0.84	0.383	0.003	0.006
Attention	-0.054±0.74	0.001±0.79	0.051	<0.0001	0.300
Language	-0.009±0.89	-0.001±0.82	0.143	<0.0001	0.028
Executive function	-0.046±0.85	-0.003±0.83	0.292	<0.0001	0.184

Values are mean±standard deviation or Nos. of participants.

aThe p-value for sex and MCI status were obtained using χ2 test.

**Figure 1:**
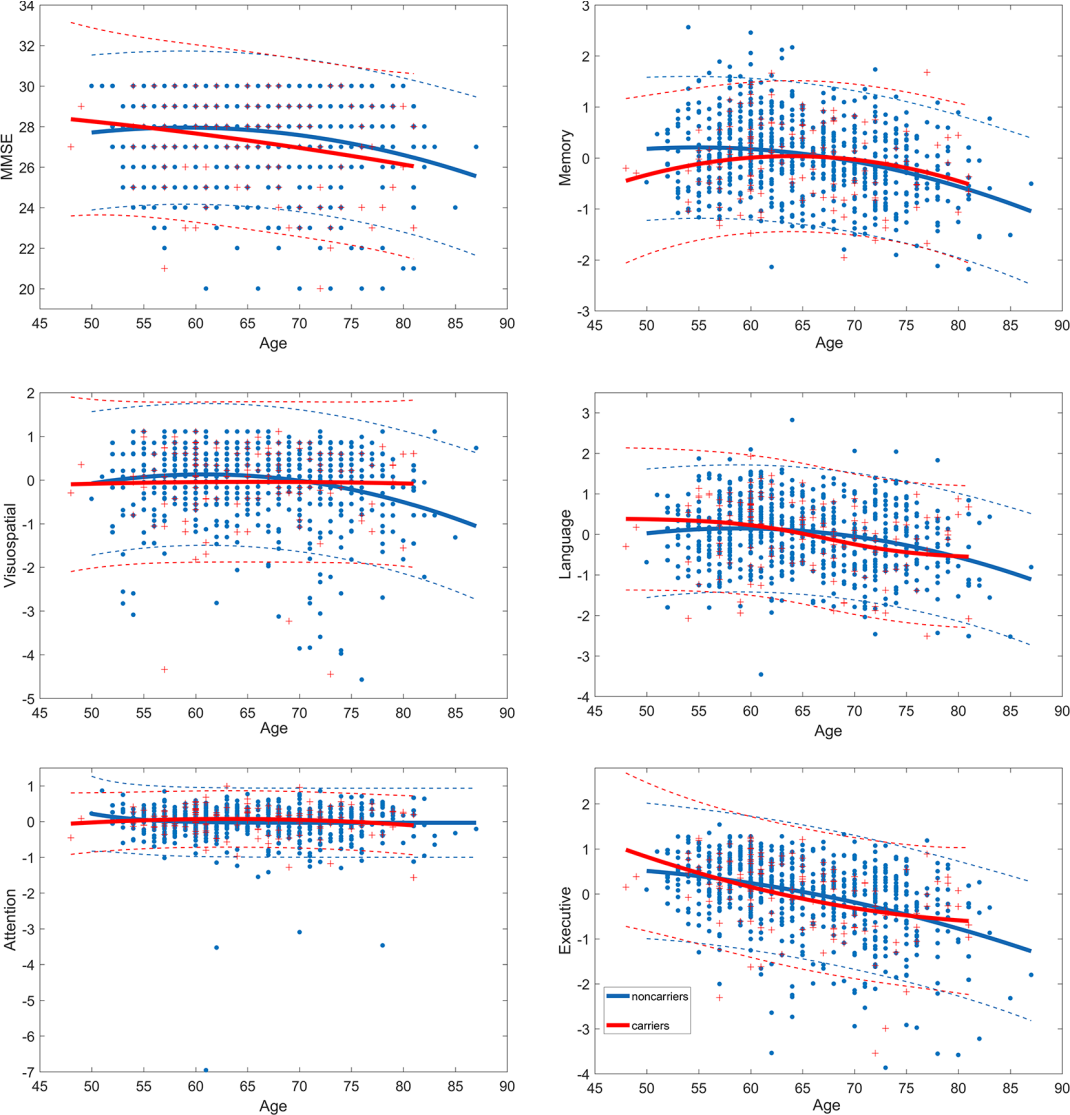
**Trajectories of age-related cognitive change in *APOE*** *ε***4 carriers and noncarriers.** Dash line represent 95% confidence intervals. MMSE, Mini-Mental State Examination.

Age-associated cognitive curves for global measures, memory, attention, visuo-spatial processing, executive function and language ability were evaluated in APOE ε4 carriers and noncarriers respectively. Best-fitting regression models were used. For carriers, age-related MMSE (regression coefficient β=-0.000458, p=0.0312), attention (β=-0.000271, p<0.0001) and executive function (β=-0.001, p<0.0001) changes tended to be quadratic rather than linear or sigmoid and language (p=0.023) tended to be sigmoid, but the effects of age on memory and visuospatial were not statistically significant in carriers (p>0.05). For noncarriers, the quadratic effect of age on MMSE (β=-0.002786, p<0.0001), memory (β=-0.001388, p<0.0001), language (β=-0.001536, p<0.0001), attention (β=-0.00061, p<0.0001), visuospatial (β=-0.00165, p<0.0001) and executive function (β=-000711, p<0.0001) with the best-fitting regression was highly significant (Figure 1). Critical age for carriers and noncarriers were identified by the extreme points of curves separately. The age-related MMSE, language, attention, and executive function decline in carriers changes steeply after age 50. However, most of the cognitive domains began to decline around age 60 in noncarriers, at the ages of 58.7 for MMSE and language, 56.3 for memory, and 60.8 for visuo-spatial processing, and attention and executive function with similar rapid changes after age 50.

### White matter integrity change with age

**Figure 2:**
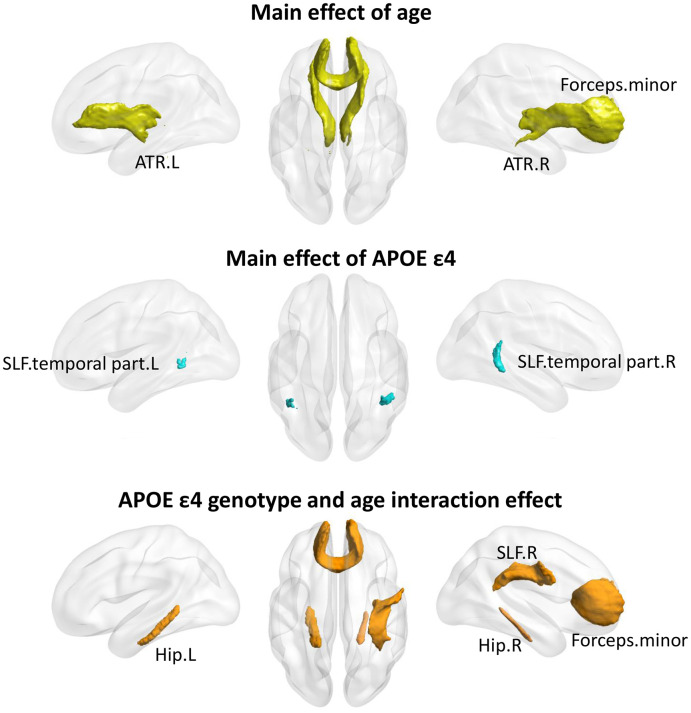
**The age×*APOEε*4 effects on fiber bundles.** Significant main effect of age (Top), main effect of *APOE* *ε*4 genotype (Middle), and age×*APOE* *ε*4 interaction (Bottom) on fibers were found (p<0.05, uncorrected). ATR, Anterior thalamic radiation; SLF, Superior longitudinal fasciculus; Hip, cingulum.hippocampus; L, left; R, right.

Based on the relatively small samples with available MRI data, there were 53 *APOE* ε4 carriers and 58 noncarriers included in white matter analysis (Supplementary Table 1). A main effect of age was found on the anterior fiber bundles, including bilateral anterior thalamic radiation (ATR), and forceps minor (FM). Significant main effect of *APOE* *ε*4 genotype were found in posterior fiber bundles, including bilateral superior longitudinal fasciculus.temporal part (Figure 2). Among the FA values at each tract, significant *APOE* *ε*4 genotype and age interaction effects were found in both anterior and posterior fibers, including bilateral cingulum. Hippocampus (Hip.L and Hip.R), FM, and right superior longitudinal fasciculus (SLF.R). Notably, the significant interaction results were not corrected for multiple comparisons and therefore should be regarded as exploratory in nature.

**Figure 3:**
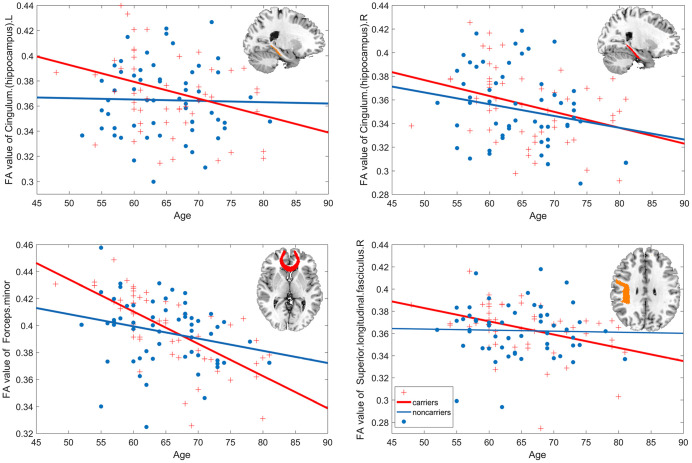
**Age-related white matter integrity decline.** The estimated age trajectories from the linear regression analyses of bilateral cingulum. hippocampus, forceps minor, and right superior longitudinal fasciculus for APOE *ε*4 carriers (red line) and noncarriers (blue line).

Further linear regression analysis showed that there was a significant age-related decline in Hip.L (carriers R^2^=0.308, noncarriers R^2^=0.224), Hip.R (carriers R^2^=0.343, noncarriers R^2^=0.179), FM (carriers R^2^=0.676, noncarriers R^2^=0.24) and SLF.R (carriers R^2^=0.394, noncarriers R^2^=0.027) in carriers, which was a slight decrease in noncarriers but not significant (Figure 3).

### Correlation between cognition and white matter integrity

**Figure 4:**
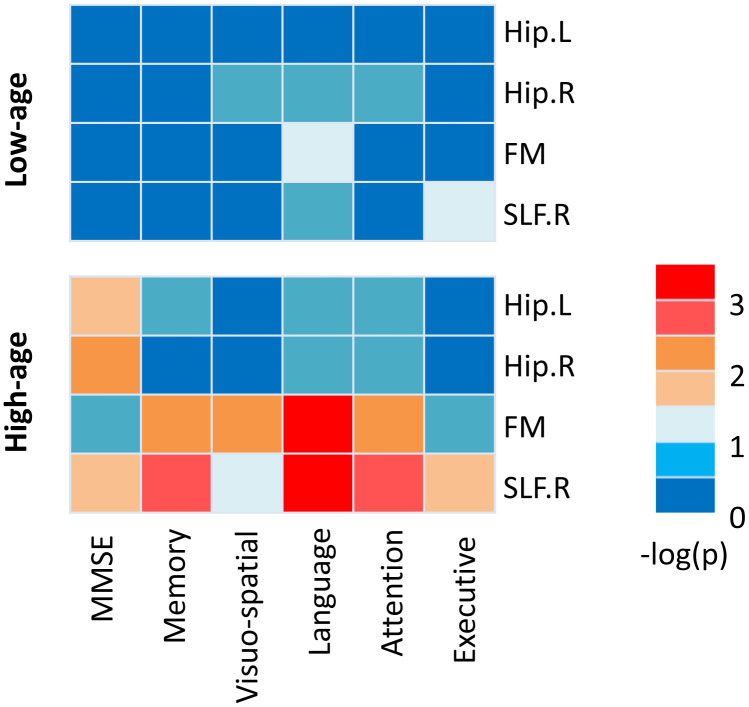
**The relationships between white matter integrity and cognitive performance in APOE ε4 carriers.** A heatmap reflecting –log (P) values for correlations between white matter integrity and cognitive performance in low-age (26 subjects, <65years) and high-age (27 subjects, ≥65years) ε4 carriers. Though a number of significant correlations were estimated high-age carriers, no correlation was significant in low-age carriers.

Correlations between each white matter integrity and cognitive score were performed in carriers in low-age and high-age group separately. As illustrated in Figure 4, within the high-age carriers, FA values of Hip.L and Hip.R were significantly correlated with MMSE (Hip.L: r=0.45, p=0.02; Hip.R: r=0.50, p=0.008), FA value of FM was significantly correlated with memory (r=0.55, p=0.005), visuo-spatial ability (r=0.56, p=0.004), attention (r=0.57, p=0.004), and language (r=0.67, p<0.001), FA value of SLF.R was significantly correlated with MMSE (r=0.48, p=0.01), memory (r=0.59, p=0.002), attention (r=0.58, p=0.003), language (r=0.63, p<0.001), and executive function (r=0.46, p=0.025). However, there is no significant relationship between white matter integrity and cognitive performance in the low-age carriers. SLF.R-language and FM-language were survived false-discovery rate (FDR) correction for multiple comparisons at a level q<0.05. Additionally, we found that significant FM-attention and SLF.R-attention correlations in low-age noncarriers and FM-language and FM-Visuo-spatial ability correlations in the high-age noncarriers (Supplementary Table 2).

## DISCUSSION

The current study found that the *APOE* ε4 allele could accelerate the age-related reductions in the general mental status, memory, language, and executive function in Chinese non-demented elderly through a large sample data. Curvilinear regression analysis found that carriers showed a steeper age-related decline after age 50 in MMSE, attention, language, and executive function and performed worse than noncarriers at almost all ages. Furthermore, the development of memory and visuo-spatial ability remained roughly low and unchanged after 50 years old in ε4 carriers. However, most of the cognitive domains began to decline around age 60 in noncarriers, at the ages of 60.1 for general mental status, 56.3 for memory, 60.8 for visuo-spatial processing, and 58.7 for language. Then, we found the interactive effect of *APOE* genotype and age on the white matter integrity of Hip.L, Hip.R, FM and SLF.R across twenty main fasciculi in the brain. Along with the increasing age, *APOE* *ε*4 carriers showed an accelerated integrity reduction of these fibers. Further analysis found significant decline of these fibers in the *APOE* *ε*4 carriers but no significant age-related changes in noncarriers. Finally, the correlation analysis found that the changed white matter integrity was associated with cognitive function in high-age ε4 carriers, but not in low-age carriers.

With simple operation and high sensitivity, the MMSE is always used as an effective tool for the detection for the basic general mental status of patients [20]. Owing to the risk of *APOE* ε4 allele, the MMSE performance in ε4 carriers was usually worse than noncarriers [21]. A previous study, stratifying participants by age in three groups (45-54, 55-64, and 65-74 years old), found a significant interactive effect of *APOE* genotype and age on MMSE, that the homozygotes performed worst in the high-age group [22]. When stratifying participants based on a median split (65 years old) in two groups, other researchers did not find any significant interaction of *APOE* genotype and age on cognition [23]. Our findings of the interaction of *APOE* genotype and increasing age on MMSE in the elderly seemed to be favor to reveal the effect of *APOE* genotype on cognition along with age. Additionally, we found *APOE* ε4 allele could accelerate the age-related reductions in the attention, executive function and language ability, which are main impaired cognitive domains in mild cognitive impairment and early stage of AD [24].

Our study also explored the trajectories of age related cognitive change affected by *APOE* genotype. The cognitive functions were almost unchanged in ε4 carriers after 50 years old. This is probably due to the effect of ε4 allele on cognitive functions is already existed before 50 years old. Multiple evidences have supported the deleterious effect of ε4 allele on memory in young adults and mice [25, 26]. It seems like that the trajectories of cognitive change in noncarriers were more similar to the process of cognitive aging [27].

As an important white matter tract connecting those cingulated subregions and projecting to the entorhinal cortex, the cingulum plays a crucial role in normal aging and AD development [28, 29] and is associated with diverse cognitive functions [30, 31]. The SLF is the longest association fiber in the brain linking bidirectionally the parietal cortex with the frontal cortex, thus providing high-order somatosensory input and information on perception of space to the frontal cortex [32]. The SLF plays an important role in memory [32]. Studies of diffusion tensor imaging (DTI) measures have demonstrated that the FA of SLF decreased in AD patients [33, 34]. The FM, connecting different prefrontal regions, could regulate attention and executive function [35, 36]. The reduced FA of FM may affect inter-hemispheric communications and may lead to sustained attention deficits [37]. In the current study we found significant interactive effects of *APOE* genotype and age on Hip.L, Hip.R, FM and SLF.R, which may suggest the neural mechanism of *APOE* on cognitive function related with increasing age. On other fasciculi including the frontal, lateral parietal, centrum semiovale, genu and splenium of corpus callosum, and temporal stem, researchers have also found significant interactive effects of *APOE* genotype and age [38]. Otherwise, some negative results have also been reported [39]. In the present study, the effect of gene - age interaction on MD was not significant. Some study showed that FA measure may be more sensitive to AD-related white matter changes than MD to some extent [40].

For the trajectories of age-related FA change, the noncarriers showed almost unchanged whereas the ε4 carriers showed accelerated age-related integrity reduction after age 50. Similar to the effect of *APOE* genotype on cognitive function, the trajectories of FA in noncarriers were like the aging processing of white matter tracts, which plateaued in the fourth decade and then declined [41]. One possible explanation for the inverted U type trajectories of FA in ε4 carriers in our study is the concept of genetic buffering, that some protective variants carried by individuals without developing disease buffering the effect of harmful variants. Some evidences from the oldest-old participants have suggested that the ε4 allele was not related with AD incident and mortality [42].

In the following correlation analysis, we categorized the participants by 65 years old and then assessed the relationship between white matter integrity and cognitive function in ε4 carriers. We found significant correlation between white matter change and cognitive function in the high-age group. Several studies have indicated that the cingulum is associated with attention [30, 31]. And the SLF is also associated with some cognitive function, like language ability [43] and working memory [44]. Studies also supported the close relationship between the FM and attention and executive function [45, 46]. Our results might suggest that these white matter tracts were distributed in pivotal regions dominant some high-order cognitive functions, thus the demyelination and axonal injury in these white matter could be the neural basis of cognitive deficits.

The APOE gene is associated with Alzheimer’s disease, but it cannot predict individual getting it. Case studies have shown tremendous progress with diet, exercise and lifestyle changes for slowing and even reversing symptoms [47, 48]. In the future, we will collect more information about these factors and pay attention to their regulation of APOE risk.

## CONCLUSIONS

In conclusion, the current study described different patterns of age related cognition and white matter integrity in *APOE* ε4 carriers and noncarriers. Our findings underscore the importance of integrating age and genetic variants when examining candidate genes for cognitive function and AD. Further prospective studies with a larger sample size and a longitudinal design would permit clarification on our findings and provide more concrete evidence.

## MATERIALS AND METHODS

### Participants

Participants were selected from the Beijing Aging Brain Rejuvenation Initiative study, an ongoing longitudinal study examining the brain and cognitive decline in an elderly, community-dwelling sample [49]. All enrolled participants were Han Chinese, right-handed. The study was approved by the Ethics Committee and institutional review board of Beijing Normal University Imaging Center for Brain Research, and written informed consent was given by all participants. To be included in this study, participants had to meet the following criteria: (1) clinically non-demented and scored at least 20 on the MMSE; (2) no history of neurologic, psychiatric, or systemic illnesses known to influence cerebral function, including serious vascular diseases, head trauma, tumor, current depression, alcoholism, and epilepsy; (3) no prior history of taking psychoactive medications; (4) a successful blood sample for the genotyping analysis. Specifically, the status “clinically non-demented” was determined by using Dementia, according to the Diagnostic and Statistical Manual of Mental Disorders IV criteria. Accordingly, 846 non-demented subjects (aged 48–87 years; 311 males/535 females) were included in the present study. We diagnosed a participant with MCI according to the previously established criteria [50].

### APOE genotyping

DNA was extracted from the subjects’ blood samples according to standard procedures for the subsequent characterization of the *APOE* genotype using PCR (Applied Biosystems, Foster City, CA). All participants were genotyped for two SNPs in the *APOE* gene (rs429358 and rs7412) using previously published methods [51]. There were 129 *APOE* ε4 carriers (including 123 ε3/ε4 genotype and 6 ε4/ε4 genotype) and 717 *APOE* ε4 noncarriers (including 602 ε3/ε3 genotype and 115 ε2/ε3 genotype) included in our present study.

### Neuropsychological testing

A comprehensive neuropsychological battery was comprised of the following 5 cognition domains with the tests included in parentheses: 1, memory (the Auditory Verbal Learning Test (AVLT), the Rey-Osterrieth Complex Figure test (ROCF) (recall), and the backward Digit Span); 2, attention (the Trail Making Test (TMT)-A, the Symbol Digit Modalities Test (SDMT) and the Stroop Color and Word Test (SCWT)-B); 3, visuo-spatial ability (ROCF (copy), Clock-Drawing Test (CDT)); 4, language (the Category Verbal Fluency Test (CVFT), the Boston Naming Test (BNT)); and 5, executive function (the TMT-B and SCWT-C). The specific neuropsychological test procedures have been described previously [52]. For each cognitive task, raw test scores were converted to z-scores. Next, composite z-scores were calculated for each cognitive domain by averaging z-scores for all cognitive tasks within a cognitive domain.

### MRI data acquisition

Among the participants, 111 subjects (53 *APOE* ε4 carriers and 58 noncarriers) received high-quality MRI scanning, which included a 3D T1-weighted MRI scan and a DTI scan. All participants were scanned with a SIEMENS TRIO 3T scanner in the Imaging Center for Brain Research at Beijing Normal University, including high-resolution T1-weighted structural MRI, and DTI. Participants laid supine with their head fixed snugly by straps and foam pads to minimize head movement. T1-weighted, sagittal 3D magnetization prepared rapid gradient echo (MP-RAGE) sequences were acquired and covered the entire brain [176 sagittal slices, repetition time (TR)=1900 ms, echo time (TE)=3.44 ms, slice thickness=1 mm, flip angle=9°, inversion time=900 ms, field of view (FOV)=256×256 mm^2^, acquisition matrix=256×256]. For each DTI scan, images covering the whole brain were acquired by an echo-planar imaging sequence with the following scan parameters: TR=9500 ms, TE=92 ms, 30 diffusion-weighted directions with a b-value of 1000 s/mm^2^, and a single image with a b-value of 0 s/mm^2^, slice thickness=2 mm, no inter-slice gap, 70 axial slices, acquisition matrix =128×128, FOV=256×256 mm^2^, averages=3.

### Diffusion MRI data processing

DTI data were processed with the FDT toolbox in FSL (https://www.fmrib.ox.ac.uk/fsl). First, the DICOM files of all subjects were converted into NIfTI using the dcm2nii tool embedded in MRIcron. Second, the brain mask was estimated, and the resulting brain mask was required for the subsequent processing steps. Third, the non-brain spaces in the raw images were cut off leading to a reduced image size, reducing the memory cost and speeding up the processing in subsequent steps. Fourth, each image was coregistered to the b0 image using an affine transformation to correct the eddy-current induced distortions and simple head-motion artifacts. The diffusion gradient directions were adjusted accordingly. Fifth, a voxel-wise calculation of the tensor matrix and the diffusion tensor metrics were yielded for each subject (i.e., the FA).

To identify major white matter tracts, we used the digital WM atlas JHUICBM-DTI-81 (http://cmrm.med.jhmi.edu/), a probabilistic atlas generated by mapping the DTI data of 78 subjects to a template image. The atlas contains 20 main WM bundles and has three individual sets of sub-templates with different probability levels in the probability tractography map: 0%, 25% and 50%. In this study, we chose to use the 25% threshold subtemplate, which contains 20 major tracts. The JHU-WM atlas was overlaid on the WM skeleton of each subject in the CBM-DTI-81 space, such that each skeleton voxel could be categorized into one of the major tracts. Then, the mean FA at the skeleton voxels within each tract was calculated.

### Statistical analysis

To evaluate the group difference (*APOE* ε4 carriers vs. noncarriers) in age and education, two-sample t-test were performed. For gender and medical history, Chi-square test was applied. For each cognitive domain, we first tested the *APOE* ε4 genotype and age interaction by using a general linear model (GLM), and then used curvilinear regression models to assess associations between each cognition and age in the *APOE* ε4 carrier and noncarrier separately. For the multiple nodal efficiencies, we applied the FDR procedure to correct the multiple comparisons at a q-value of 0.05.

### Associations between cognitive function and age

We assessed the *APOE* ε4 genotype × age interaction on each cognitive domain (raw MMSE score or composite cognitive domain scores). Specifically, a GLM with “age”, “*APOE* ε4” and “*APOE* ε4 × age” as predictor variables were applied, in which gender and education were included as covariates. Curvilinear regression models were used to assess associations between cognitive functions and age in the *APOE* ε4 carriers and noncarriers respectively. We use linear, quadratic, or sigmoidal regression curve to fit different cognitive functions and judge from the goodness of fit to data (R^2^).

### Fiber diffusion metrics change with age

The influence of the *APOE* ε4 × age interaction on fiber diffusion metrics (i.e., the FA) at each tract was tested using GLM. Specifically, for each fiber, we applied a GLM with “age”, “*APOE* ε4” and “*APOE* ε4 × age” as predictor variables. Also, gender and education were included as covariates.

### Age effect on correlation between fiber diffusion metrics and cognitive functions

We further investigated how the age influences correlation between the above-identified white matter tracts and the cognitive performance in *APOE* ε4 carriers and noncarriers separately. Since there is no easy way to plot a two-way interaction with two continuous variables (i.e., white matter integrity and age), we first equally divided all carriers or noncarriers into four sub-groups in terms of the rank of age: low-age carriers (26 carriers, <65 years) and high-age carriers (27 carriers, ≥65 years), low-age noncarriers (30 noncarriers, <65 years) and high-age noncarriers (28 noncarriers, ≥65 years). And then calculate the partial correlation of white matter integrity and cognitive scores, accordingly. Gender and education were included as covariates.

## Supplementary Material

Supplementary Tables
