# Supplementary material for: APOE ε4 allele accelerates age-related multi-cognitive decline and white matter damage in non-demented elderly
Source: Aging (Albany NY). 2020 Jun 22;12(12):12019–31. doi: 10.18632/aging.103367 (PMC7343443; doi:10.18632/aging.103367)
Supplement: Supplementary Tables [file aging-12-103367-s001..pdf]

SUPPLEMENTARY TABLES

Supplementary Table 1. Demographic and neuropsychological tests of white matter analysis sample.

	APOE ε4 carriers(n=53)	*APOE ε4* noncarriers (n=58)	Main *APOE ε4* effect	Main age effect	Age×*APOE ε4* interaction
Male/Female	22/31	29/29	0.45[a]		
Age (years)	65.53±7.71	64.72±6.52	0.55		
Education (years)	11.79±3.08	11.07±3.37	0.24		
General mental status	27.19±2.48	27.74±1.61	0.39	0.12	0.45
Memory	0.088±0.84	-0.061±0.66	0.63	0.02	0.68
Visuo-spatial ability	0.042±0.79	0.095±0.64	0.80	0.002	<0.0001
Attention	0.051±0.73	-0.006±0.71	0.52	0.23	0.36
Language	0.124±0.85	0.022±0.77	0.70	0.103	0.06
Executive function	0.139±0.63	-0.106±0.91	0.735	0.201	0.61

Values are mean±standard deviation or Nos. of participants.

Supplementary Table 2. Correlation between cognition and white matter integrity in noncarriers.

			Hip.L	Hip.R	FM	SLF.R
Low-age noncarriers	MMSE	r	0.187	0.229	-0.217	-0.001
		p	0.352	0.250	0.277	0.996
	memory	r	0.064	-0.013	-0.234	-0.118
		p	0.750	0.949	0.241	0.558
	Visuo-spatial	r	0.139	0.242	-0.010	0.216
		p	0.491	0.224	0.962	0.280
	language	r	-0.068	0.127	0.197	0.288
		p	0.737	0.528	0.326	0.145
	attention	r	0.078	0.204	**0.572**	**0.447**
		p	0.698	0.308	**0.002**	**0.019**
	executive	r	0.197	0.057	0.370	0.367
		p	0.326	0.779	0.057	0.060
High-age noncarriers	MMSE	r	-0.193	-0.330	0.389	0.081
		p	0.367	0.115	0.060	0.705
	memory	r	-0.137	-0.179	0.215	-0.229
		p	0.522	0.404	0.312	0.281
	Visuo-spatial	r	-0.097	-0.131	**0.706**	0.276
		p	0.652	0.543	**<0.0001**	0.191
	language	r	-0.095	-0.188	**0.515**	0.095
		p	0.659	0.378	**0.010**	0.658
	attention	r	0.153	-0.296	0.374	-0.087
		p	0.474	0.161	0.072	0.686
	executive	r	0.072	-0.383	0.072	-0.180
		p	0.739	0.064	0.739	0.401
